# Evidence for multiple motivational accounts of willful ignorance in prosocial decision making beyond moral wiggling

**DOI:** 10.1038/s41598-026-59730-1

**Published:** 2026-07-03

**Authors:** Fiona tho Pesch, Anna Baumert, Susann Fiedler

**Affiliations:** 1 Department of Psychology, University of Cologne, Cologne, Germany; 2 Max Planck Institute for Research on Collective Goods, Bonn, Germany; 3 Department of Social and Human Sciences, University of Wuppertal, Wuppertal, Germany; 4 Department of Strategy and Innovation, Institute for Cognition and Behavior, Vienna University of Economics and Business, Vienna, Austria

**Keywords:** Willful ignorance, Moral wiggle room, Prosociality, Interindividual differences, Psychology, Human behaviour

## Abstract

People are generally expected to seek information, especially when it informs their decisions. As such, the phenomenon of willful ignorance—avoiding behaviorally relevant information—remains puzzling. This research explores plausible motivations behind willful ignorance in prosocial decision making, a behavior with negative societal implications. While it is traditionally attributed to selfish motives (moral wiggling), alternative explanations like tradeoff aversion and inattention have received little attention. In a within-subject design (*N* = 878), we examined behavioral patterns across decision contexts designed to render specific kinds of motivations more or less plausible. Specifically, we compare decision shifts when the option to ignore is given vs not given across contexts that systematically vary the plausible motivational relevance of ignorance. Our results suggest that approximately one fifth of ignorance cases are consistent with moral wiggling, another fifth with tradeoff aversion, and roughly one third with inattention. In addition, about one sixth of ignorance cases occurred in situations in which the information was not instrumental to participants’ decisions. These findings challenge the traditional view that ignorance is mainly selfishly motivated, underscoring the complexity of human behavior. By highlighting the possibility of multiple motivational accounts of willful ignorance, this study calls for a nuanced approach to mitigating its societal consequences.

**Supplementary Information:**

The online version contains supplementary material available at 10.1038/s41598-026-59730-1.

## Introduction

The idea that knowledge is power^1^ has been widely accepted, yet research has demonstrated that people ignore crucial and freely available information that would otherwise be instrumental in their decision making^2^. For example, though they care about animal welfare, some people ignore information regarding meat production when consuming meat products^3^, or people who care about the environment ignore information on environmental pollution when choosing plane tickets^4^. Similarly, in standardized economic game settings, people have been shown to avoid information that would compel them to behave prosocially, and subsequently they choose more selfishly^5^. The predominant explanation for this behavioral pattern has been that people want to reap the benefits of choosing a self-serving option without appearing selfish, either to others or themselves. In the present paper, we scrutinized this explanation by testing it against plausible alternative motivational mechanisms. Exploring the potential motivational foundations of willful ignorance through observed behavioral patterns provides insight into possible drivers of prosocial behavior and contributes to a broader understanding of social decision-making.

Most research on willful ignorance in prosocial decision making has used the *hidden information treatment*^5^, in which participants are asked to make a binary dictator game decision on how to distribute money between themselves and another participant. Results consistently showed that participants are less likely to be generous when they can ignore the payoffs to the other participant^5–10^ (for a review see^11^).

The prevalent explanation for the effect of willful ignorance on prosocial behavior is that individuals wish to maximize their own benefits without appearing selfish (i.e., exploiting *moral wiggle room*): By willfully ignoring the consequences of their behavior, agents can plausibly claim that they would have acted virtuously if they had been informed, meaning that they can still uphold a positive self- and social image^5–9,12–14^. In the following, we will refer to these motives as wiggling-related motivations.

Notably, several studies have provided suggestive evidence that other motivations could potentially be involved in the willful ignorance effect. For example, some people were found to ignore their *own* payoffs when these were initially hidden (instead of the interaction partner’s payoffs) and could be revealed only by clicking a button^15,16^. Other studies found that willful ignorance extended beyond self-other tradeoffs and occurred also when individuals made decisions on behalf of others^17^, or distributed money as a third party^18^. Together, these results pose a puzzle as to which motivations drive willful ignorance. In a meta-analysis of willful ignorance, Vu et al.^11^ discuss potential alternative mechanisms, such as inattention. They conclude that ignorance is mainly driven by selfishness, based on the argument that people who ignore are more likely to choose the self-serving option. Here, we argue that this conclusion is premature. Given the structure of the decision space within the hidden information treatment^5^, the self-serving option becomes the strictly dominant decision as long as the payoffs to the other participant are not revealed. In other words, participants have no plausible reason to choose the option with the lower payoff for themselves as long as they stay ignorant. As such, ignorance leads to more selfish decisions, even if the motives for remaining ignorant are unrelated to a desire to act selfishly while preserving one’s image, such as in cases of inattention. To further explore these mechanisms, we conducted a high-powered, preregistered experiment employing a repeated-measures design that allowed us to assess patterns consistent with different motivational accounts of ignorance choices.

Besides wiggling-related motivations, in the literature, we found discussions of two potential alternative motivational mechanisms of willful ignorance: tradeoff aversion and inattention. Tradeoff aversion refers to the idea that people may prefer not to make a decision in certain situations. Such a preference is at odds with the general assumption that decision opportunities are desirable because they increase the chances of finding an option that aligns with one’s preferences^19^. Notably, decision opportunities can lead to cognitive load, and trigger negative affective states such as feeling tempted, fear of making a bad decision and eventually regret^20,21^. In the context of the hidden information treatment by Dana et al.^5^, participants can avoid having to make a tradeoff decision by choosing to stay ignorant. This is because under ignorance one option is strictly dominant to the other. In the hidden information treatment, the dominant option happens to be the selfish option. So a desire to avoid making a tradeoff would lead to more selfish decisions, even if the individual is not genuinely motivated to hide selfish motives (i.e. exploiting wiggle room). Accordingly, previous studies using the hidden information treatment might have overestimated the relevance of wiggling-related motivations^18^.

A second alternative mechanism is inattention. People may remain ignorant due to a general lack of attention within the decision context, possibly because they feel overwhelmed or uninterested. As also noted by Vu et al.^11^, inattention may lead to inaction, meaning that individuals remain ignorant in the hidden-information treatment. This, in turn, may result in participants simply choosing the option with the highest payoff under ignorance, which in the classic setup of Dana et al.^5^ corresponds to the selfish option. Consequently, inattention—much like motivated accounts of inaction such as wiggle-related motives or tradeoff aversion—can increase selfish choices in this paradigm even in the absence of selfish motives. This dynamic may therefore lead to an overestimation of wiggle-related motivations as drivers of the willful ignorance effect.

In our study, we scrutinize behavioral patterns across different decision contexts, which make each of the three motivations (i.e., wiggling, tradeoff aversion and inattention) more or less plausible. We assessed the potential contribution of each motivation by having participants make allocation decisions in three distinct types of binary dictator game contexts. Our within-subject design allows us to examine how decisions change between a baseline, where all payoffs are revealed, and a hidden-information condition, where participants can choose to remain ignorant about some payoffs, across different contexts. It also allows us to analyze participants’ choices to engage in ignorance across these contexts. By combining this within-subject manipulation of decision context with interindividual differences measures, our research substantially extends prior empirical approaches^18^. While Exley and Kessler^18^ also varied the decision context of ignorance decisions, their between-subject design did not allow for further exploration in terms of what motivations plausibly drove ignorance choices. By contrast, our approach allows us to investigate an individual’s choice patterns across different decision contexts, and predict these behavioral patterns from interindividual dispositional differences. As such, our design is better equipped to shed light on the plausible motivational foundations of willful ignorance.

**Fig. 1:**
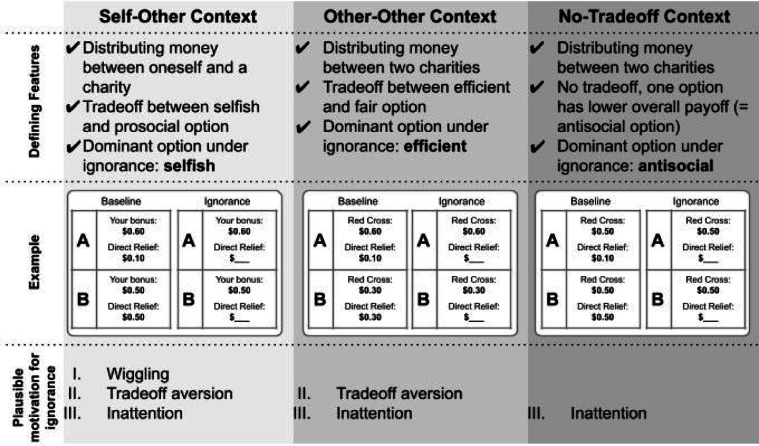
Overview of decision contexts.

To this end, we designed three different decision contexts: the Self-Other context, the Other-Other context, and the No-Tradeoff context (see Fig. 1). Each context included a baseline and an ignorance condition. In the latter, some payoff information was initially hidden (i.e., parallel to the hidden information condition of Dana et al.^5^). Note that participants also made decisions in two additional decision contexts which will not be discussed within this paper. For results on these contexts, and a rationale for excluding them from the main text, see Supplemental Materials. Across four waves of data collection, participants made two allocation decisions per context (i.e. one within the ignorance setting, and one within the baseline setting).

In the Self–Other context, participants decided how to allocate money between themselves and a charity by choosing between a selfish and a prosocial option. In the baseline condition, all payoffs were visible. In the ignorance condition, the charity’s payoff was initially hidden, and participants could choose whether to reveal it before deciding. Participants knew that in 50% of the cases the payoff structure was identical to the baseline condition (i.e. one option would be selfish, the other prosocial), while in the other 50% it was flipped (i.e. the option favoring the participant would also favor the charity). Thus, under ignorance, participants knew which option maximized their own payoff and they knew there was a 50% chance that this option would result in a lower donation to the charity.

In the Other–Other context, participants allocated money between two charities. In the baseline condition, they chose between a fair option and an efficient option. In the ignorance condition, the payoff for one of the charities was initially hidden. Again, participants knew that in 50% of the cases the payoff structure was identical to the baseline condition (i.e. one option would be efficient, the other fair), while in the other 50% it was flipped (i.e. the efficient option would also be the fairer option). Under ignorance, participants knew which option was the efficient option and they knew there was a 50% chance that this option would result in a lower donation to the second charity.

In the No-Tradeoff context, participants again allocated money between two charities. The first charity’s donation was fixed, independent of the decision, while the second charity received more in one option than in the other, making one option strictly dominant. In the ignorance condition, payoffs to the second charity were initially hidden.

This design allowed us to systematically alter the motivations that could plausibly be driving willful ignorance in each specific context. In the Self-Other context, by design, there are three plausible motivators for willful ignorance: wiggling, tradeoff aversion, and inattention. Contrasting the Self-Other to the Other-Other context, we eliminate wiggling as a plausible motivation because the participant’s own payoff is not at stake in this decision. While the decision in the Other-Other context still involves a tradeoff between an efficient and a fair option, the No-Tradeoff context removes tradeoff aversion as a plausible motivation, leaving inattention as a plausible reason for willful ignorance.

Note that, across decision contexts, allocation decisions varied qualitatively (selfish vs. prosocial, efficient vs. fair, and dominant vs. antisocial). Thus, we refrained from directly comparing allocation decisions across contexts. Instead, we compared ignorance levels between these three contexts, and we compared how the option to ignore (i.e. baseline vs ignorance condition) shifted participants’ allocation decisions. Inspecting within-person decision shifts and ignorance decisions allowed us to infer the theoretically plausible impact of wiggling-related motives, tradeoff aversion and inattention.

To this end, we hypothesized that overall, participants would be more likely to choose the option with the higher payoff under ignorance when the option to stay ignorant was present (or in case of the No-Tradeoff setting, the option with the lower payoffs under transparency). This means that in the Self-Other-ignorance condition, participants would be more likely to choose the selfish option, in the Other-Other-ignorance condition, they would be more likely to choose the efficient option, and in the No-Tradeoff-ignorance condition, they would be more likely to choose the antisocial option as compared to their respective baselines. This serves as a proof of concept, because if it did not hold, it would imply that only individuals indifferent to the hidden information choose to remain ignorant.

By comparing the effect of the option to remain ignorant on decision behavior in three decision contexts, we can see in which way the effect of willful ignorance is unique to prosocial decision contexts (Goal 1). Assuming that all three kinds of motivations could play a role, we predicted that the impact of the ignorance manipulation on behavior would decrease from the Self-Other to the Other-Other to the No-Tradeoff context. In the same vein, we predicted that participants would choose to stay ignorant more often in the Self-Other-ignorance condition compared to the Other-Other-ignorance condition and again compared to the No-Tradeoff-ignorance condition. Importantly, these analyses test average directional effects of the experimental manipulations—that is, whether the availability of ignorance systematically shifts decisions in the predicted direction across participants. They are not intended to capture fully individualized motivational trajectories, but to show whether the hypothesized effects emerge at the population level.

As a further unique strength of our study, observing multiple (allocation and ignorance) decisions from the same participant allows us to identify intra-individual decision patterns across contexts: We can determine who ignores in which context, and how this affects their allocation decisions, identifying *types* of ignorance and decision patterns (Goal 2). These intra-individual patterns can be linked to interindividual differences in dispositional characteristics (Goal 3) to highlight who ignores under which conditions, allowing to infer motivational reasons.

## Results

### Goal 1: How unique is willful ignorance to prosocial decision making? Comparing allocation and ignorance decisions between decision contexts

All regression analyses are conducted in a repeated-measures framework that accounts for the non-independence of observations within participants. Regression models estimate average directional effects of the experimental manipulations while incorporating subject-level random intercepts, ensuring that reported effects are informed by within-person variation across conditions rather than purely between-subject differences.

### Allocation decisions

In a repeated-measurement logistic regression framework, we regressed allocation decisions (0 = option with lower payoff under ignorance, 1 = option with higher payoff under ignorance) on ignorance condition (0 = baseline, 1 = ignorance condition), decision context (Self-Other, Other-Other and No-Tradeoff, Other-Other context as reference category) and the interaction terms of ignorance condition and decision context.

**Fig. 2:**
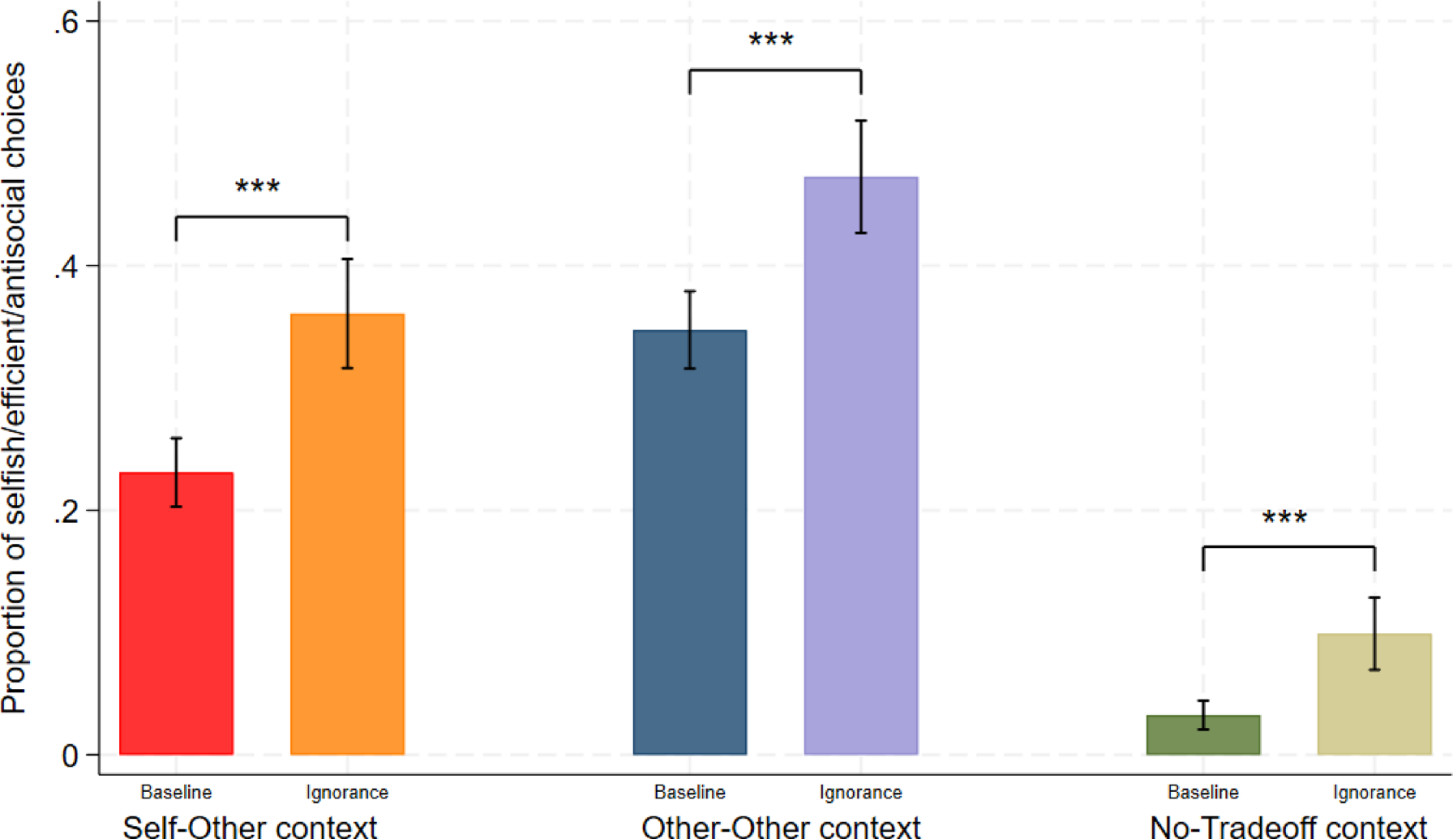
Allocation decisions in the ignorance vs baseline conditions within the Self-Other context (left), the Other-Other context (center), and the No-Tradeoff context (right) with *** indicating p < 0.001.

The results showed that, across contexts, participants were more likely to choose the allocation option with the higher payoff, or in case of the No-Tradeoff setting, the option with the lower total payoffs under transparency (i.e., the selfish, efficient, or antisocial option, respectively) when given the opportunity to ignore parts of the payoffs, compared to the respective baselines, OR = 2.00, 95% CI [1.52, 2.63] (see Fig. 2).

Furthermore, there was a significant interaction effect of ignorance condition and decision context, indicating that the effect of ignorance on allocation decisions was more pronounced in the Other-Other context (12.52% decision shift) compared to the No-Tradeoff context (5.74% decision shift), OR = 1.98, 95% CI [1.09, 3.61]. There was no significant difference between the effects of ignorance on allocation decision between the Self-Other context and the Other-Other context (12.09% vs. 12.52%), OR = 1.12, 95% CI [0.75, 1.66].

### Decision to ignore

**Fig. 3:**
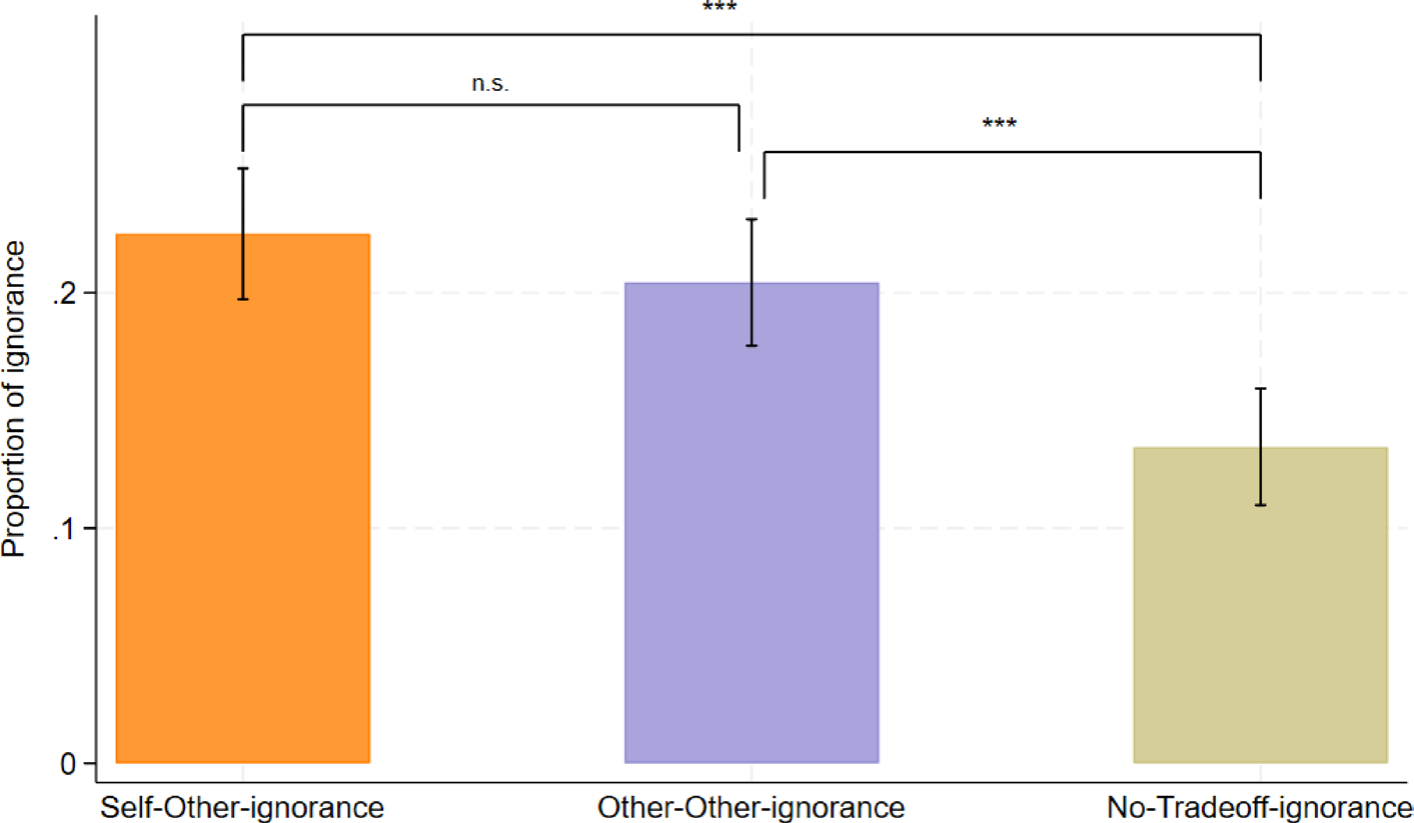
Decisions to stay ignorant (only ignorance conditions) for the different decision contexts with *** indicating p < 0.001.

Zooming in on the ignorance conditions, we regressed the decision to stay ignorant (0 = reveal, 1 = ignore) on decision context (Self-Other, Other-Other and No-Tradeoff, No-Tradeoff as reference category). Participants were less likely to ignore in the No-Tradeoff-ignorance condition (13.45%) compared to the Self-Other-ignorance condition (22.52%), OR = 3.55, 95% CI [2.40, 5.24], and the Other-Other-ignorance condition (20.44%), OR = 2.78, 95% CI [1.89, 4.09]. There was no significant difference in the likelihood of ignoring between the Self-Other- and Other-Other-ignorance condition, OR = 1.28, 95% CI [0.92, 1.76] (see Fig. 3).

### Goal 2: Typologies

Capitalizing on our within-subject design, we identified different intra-individual decision patterns across contexts. First, we identified patterns that plausibly indicate exploiting moral wiggle room by comparing allocation decisions in the Self-Other context between the baseline and the ignorance condition: 60.71% of our sample were consistently prosocial across the Self-Other-baseline and the Self-Other-ignorance condition, and 18.97% were consistently selfish. 16.96% of our sample behaved as *moral wigglers*, that is, they chose prosocially in the Self-Other-baseline and selfishly in the Self-Other-ignorance condition (3.35% showed the reverse pattern). Of these moral wigglers, 73.68% had ignored in the Self-Other-ignorance condition.

**Fig. 4:**
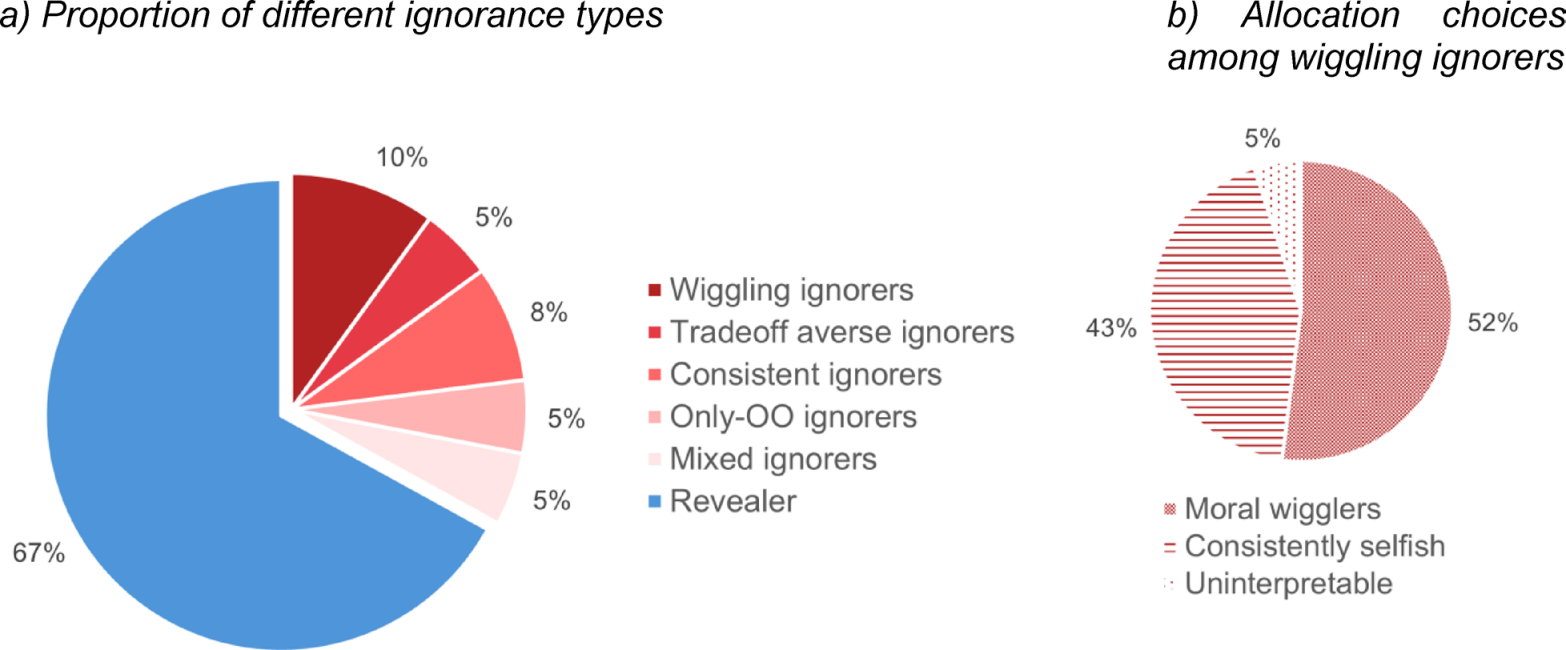
(**a**) Wiggling ignorers = only ignore in Self-Other-ignorance conditions; Tradeoff averse ignorers = only ignore in Self-Other- and Other-Other-ignorance condition; Consistent ignorers = ignore in all three ignorance conditions; Only-OO ignorers = only ignore in Other-Other-ignorance condition; Mixed ignorers = ignore at least once, but do not fit any of the other specified types. (**b**) Consistently selfish = Wiggling ignorers who chose selfish in both Self-Other-baseline and Self-Other-ignorance condition; Moral wigglers = Wiggling ignorers who chose prosocial in the Self-Other-baseline, but selfish in the Self-Other-ignorance condition; Uninterpretable = Wiggling ignorers who do not fit any of the other specified types.

Second, we investigated intra-individual patterns of ignorance decisions across decision contexts. 66.94% of participants revealed all payoffs in all three contexts (*consistent revealers*) while 33.06% of participants ignored at least once (*ignorers*, see Fig. 4). Amongst the *ignorers*, 29.83% ignored only in the Self-Other-ignorance condition (*wiggling ignorers*); 13.87% in the Self-Other- and the Other-Other-ignorance condition (*tradeoff averse ignorers*), while 24.37% ignored in all three conditions (*consistent ignorers*). 15.97% of the participants who chose to ignore did not fit into any of the previous patterns (*mixed ignorers*), and another 15.97% ignored only in the Other–Other condition—a group not captured by our predefined patterns, yet still representing a substantial portion of the sample. When further investigating the allocation decisions of the *wiggling ignorers* in the Self-Other context, we saw that 43% were consistently selfish, while 52% displayed a pattern plausibly indicating exploiting moral wiggle room: They chose selfishly only in the ignorance condition, but prosocially in the baseline condition (5% chose the low payoff option under ignorance, see Fig. 4).

In turn, we determined what percentages of the ignorance decisions observed in the Self-Other-ignorance condition was attributable to each ignorance type (wiggling ignorer, tradeoff averse ignorer, consistent ignorer). We found that 40.80% of ignorance decisions in the Self-Other-ignorance condition were made by participants classified as *wiggling ignorers*, 18.87% by participants classified as *tradeoff averse ignorers* and 33.33% by participants classified as *inattentives*.

### Goal 3: Dispositional measures

As a further approach to understanding the motivational basis for the observed decisions, we predicted allocation and ignorance decisions in the different decision contexts by means of dispositional characteristics that are conceptually linked to the motivations in question (for an overview, see Supplemental Materials). In a nutshell, Social Value Orientation (SVO)^22^, Honesty-Humility^23^, Fear of Negative Evaluation^24^, and Guilt Proneness^25^ capture interindividual differences in social concerns, Desirability of Control^26^, and Need for Closure^27^ capture one’s tradeoff aversion, and Need to Evaluate^28^ and Conscientiousness^23^ capture one’s dispositional attentiveness.

### Predicting ignorance decisions

**Fig. 5:**
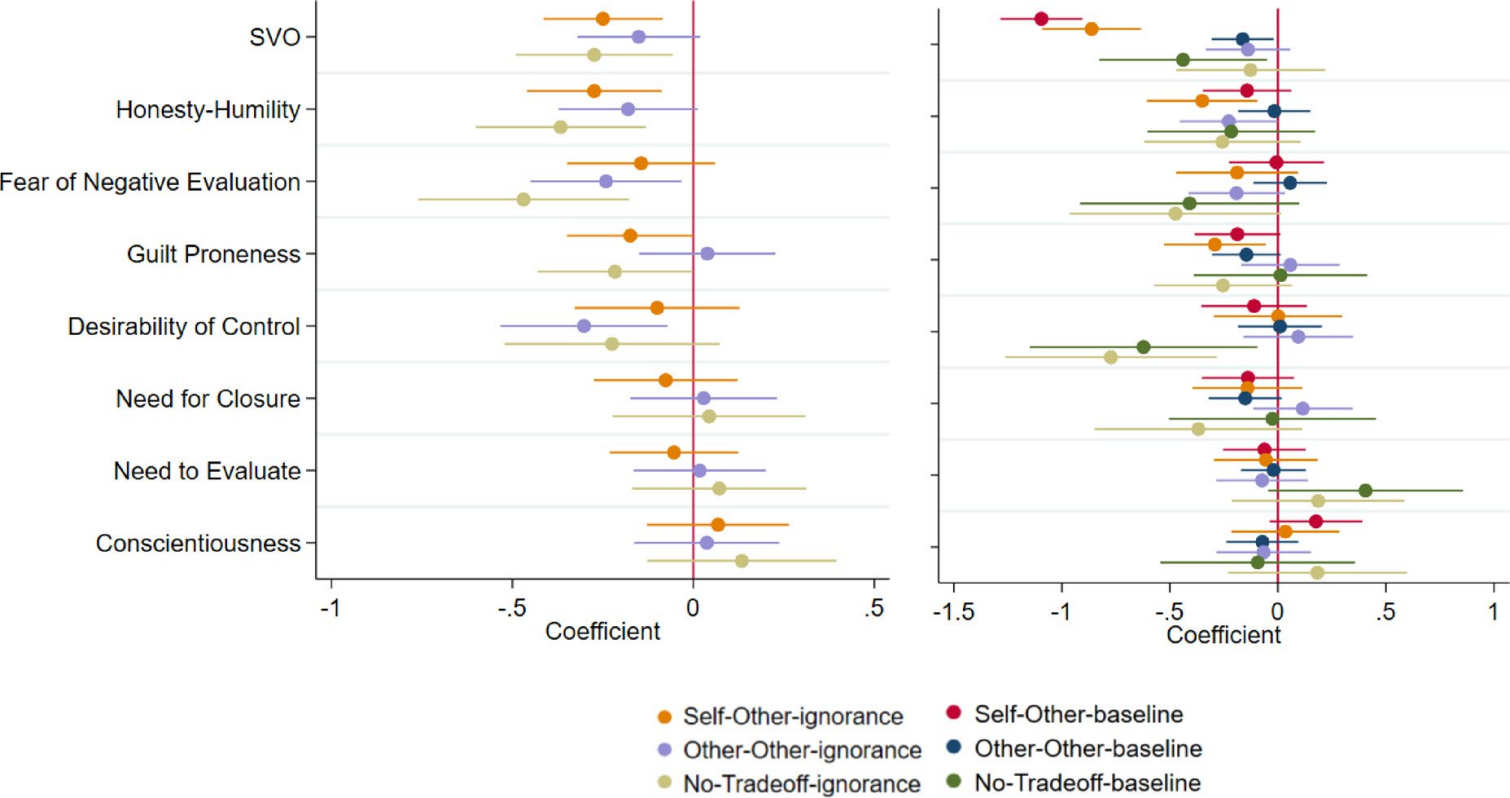
Standardized beta coefficients (and their confidence intervals) from logistic regressions predicting ignorance decisions (0 = reveal, 1 = ignore) and allocation decisions (0 = prosocial/fair/maximizing, 1 = selfish/efficient/antisocial) with dispositional measures.

Separately for decision contexts, we regressed the ignorance decision (0 = reveal, 1 = ignore) on eight dispositional measures as simultaneous predictors. We found that lower scores on SVO and Honesty-Humility predicted Self-Other-ignorance; lower scores on Fear of Negative Evaluation and Desire for Control predicted Other-Other-ignorance; and No-Tradeoff-ignorance was predicted by lower scores on SVO, Honesty-Humility, Guilt Proneness, and Fear of Negative Evaluation (see Fig. 5, for all statistics, see Supplemental Materials).

### Predicting allocation decisions

To explore the relationship between our eight dispositional measures and allocation decisions across different contexts, we conducted six separate logistic regressions—one for each condition—using jointly all eight dispositional measures as predictors (for all statistics, see Supplemental Materials).

In the Self-Other-baseline, we replicated the well-established negative link between SVO and selfish decisions^29^. In the Self-Other-ignorance condition, SVO, Honesty-Humility, and Guilt Proneness all negatively predicted selfish decisions. In the Other-Other-baseline, SVO negatively predicted efficient decisions. In the Other-Other-ignorance condition, only Honesty-Humility significantly positively predicted efficient decisions. In the No-Tradeoff-baseline condition, SVO, and Desirability of Control, negatively predicted antisocial decisions. In the No-Tradeoff-ignorance condition, only Desirability of Control significantly negatively predicted (antisocial) decisions.

### Linking types and dispositions

Linking dispositional characteristics to the identified ignorance and allocation types can provide insight into the possible motive and value structure of each group. To this end, we ran several ANOVAs combined with post-hoc t-tests investigating differences in our dispositional measures between types.

Interestingly, when examining ignorance types, both *consistent revealers* as well as *only-OO ignorers* were more prosocial as measured by SVO and Honesty-Humility compared to *wiggling ignorers*, all *p*s < 0.045. This higher dispositional prosociality is also mirrored in higher proportions of prosocial choices amongst these groups, all *p*s < 0.018 (for all related analyses, see Supplemental Materials).

Regarding allocation types, we found a significant difference between the three groups of *consistent prosocials*, *consistent selfish* and *moral wigglers* in terms of their SVO scores, *F*(2, 429) = 71.05, *p* < 0.001. Participants classified as *moral wigglers* had lower SVO scores compared to the consistent prosocials, *t*(345) = 4.04, *p* < 0.001, but higher SVO scores than the consistently selfish participants,* t*(159) = -5.81, *p* < 0.001. The same pattern appeared for Guilt Proneness, all *p*s < 0.001. We also found differences between these groups with regards to Honesty-Humility: Consistent prosocials showed higher scores than moral wigglers, *t*(345) = 4.89,* p* < 0.001, but there was no significant difference between consistent selfish types and moral wigglers, *t*(159) = 1.20, *p* = 0.231. We found no significant differences between these groups in the Fear of Negative Evaluation, Desirability of Control, Need for Closure, Need to Evaluate, or Conscientiousness, all *p*s > 0.44.

## Discussion

In the present study, we explored potential motivations of willful ignorance in prosocial decision making. Specifically, we examined the plausibility of moral wiggling motives, meaning that people choose to ignore information in order to act selfishly without appearing selfish. We used variations of the classic hidden information design^5^ in a large-scale multiple-wave study and manipulated the decision context and the option to stay ignorant within-subject. By varying specific details of the decisions, we created three decision contexts intended to make different motivations for engaging in ignorance more or less plausible, while keeping the general structure of the decision space constant. By observing behavioral patterns across all three contexts, we aim to shed light on the types of motives that could potentially contribute to ignorance in the classic hidden information paradigm^5^.

Overall, results showed that ignorance was relevant across all three contexts. Participants were most likely to ignore in the Self-Other context (22.52% of participants), where they distributed money between themselves and a charity. In the Other-Other context, where decisions did not affect own outcomes and involved a decision between fairness and efficiency, the likelihood to ignore (20.44% of participants) was similar to the Self-Other context. The lowest ignorance occurred in the No-Tradeoff context (13.45% of participants), where decisions did not affect own outcomes and one option was clearly dominant under transparency. Crucially, the option to ignore influenced allocation decisions across all contexts: Within the Self-Other context, participants were less likely to choose prosocially when they had the option to ignore (12.09% decision shift). The option to ignore had a similar effect on allocation decisions in the Other-Other context (12.52% decision shift), while being weaker in strength in the No-Tradeoff context (5.74% decision shift). Notably, the shift observed in the Self-Other context replicated the main finding of Dana et al.^5^ and aligned with findings from a recent meta-analysis, which estimated that the option to ignore leads to a decrease of prosocial behavior by approximately 15.6 percentage points^11^.

Taken together, these results suggest that some ignorance can plausibly be attributed to moral wiggling, but that it is unlikely to be the only motivation underlying willful ignorance. Remember that the structure of the decision space in the Self-Other context (which copies the classic hidden information treatment by Dana et al.^5^) is such that, ignorance—independent of how it is motivated—will likely lead to self-serving choices as these are strictly dominant under ignorance. In our study, the Other-Other decision context and the No-Tradeoff decision context were designed to reduce the plausibility of certain motivations for ignorance: In the Other-Other context, wiggling-related motives are implausible explanations for ignorance, and in the No-Tradeoff context, neither wiggling nor tradeoff aversion are plausible motivations for ignorance. Nevertheless, we observed ignorance in these contexts as well, and ignorance affected allocation choices to some extent.

Thus, the behavioral patterns that we observed in our data suggest that ignorance in the Self-Other context could potentially reflect several theoretically plausible motivations, including wiggling-related considerations, a general reluctance to engage in trade-off decisions, and inattention. This general conclusion that ignorance is consistent with several motivational accounts is supported by our analyses of within-person patterns of allocation and ignorance decisions, and the quantification of resulting allocation and ignorance types. Focussing on allocation decision patterns within the Self-Other context, we found 60.71% of our sample to be consistently prosocial, while 18.97% were consistently selfish. Importantly, 16.96% of our sample chose the prosocial option in the baseline condition, while choosing selfishly when they could ignore the consequences of their decision for the charity. Thus, a subset of participants showed a pattern previously interpreted as exploiting moral wiggle room. However, our design allowed us to further assess how this specific pattern of allocation choices was related to ignorance choice in the different decision contexts, thus scrutinizing whether this behavior was actually fully consistent with wiggling motives—ignoring information to maximize personal gain without appearing selfish.

To this end, we further investigated participants’ ignorance choices across our three different decision contexts. Our results indicate that 24% of those who ignored at least once, ignored across all contexts, suggesting inattentiveness or disinterest rather than strategic avoidance. Furthermore, 14% ignored in the Self-Other and Other-Other contexts but not the No-Tradeoff context, potentially indicating some form of tradeoff aversion. Importantly, 30% ignored exclusively in the Self-Other context, a behavioral pattern that aligns with wiggling-related motives, a group that we call *wiggling ignorers*.

To assess the extent to which willful ignorance was plausibly driven by a desire to act selfishly without appearing selfish, we examined participants in the Self-Other-ignorance condition who ignored information exclusively in that context—these so-called *wiggling ignorers*. These individuals accounted for 40.80% of ignorance choices within the Self-Other context. Of those *wiggling ignorers*, 52% also engaged in wiggling behavior—choosing prosocially in the baseline but selfishly when ignorance was possible. Another 43% of those *wiggling ignorers* consistently chose the selfish option in both cases. We can speculate that these individuals may have ignored either to avoid feeling bad or because the information was irrelevant to their choice. Excluding the latter group, only 21.22% of those who ignored in the Self-Other context displayed behavioral patterns plausibly in line with strategic, wiggling-related motives. We also observed behavioral patterns that seem more consistent with inattention (33.3%) or tradeoff aversion (18.9%) as plausible motivators, reflecting the complexity of this behavior. Note that, if we were to apply the simpler calculation method used by Exley and Kessler^18^ in their between-subject design, this would yield in our data a lower estimate of 9.23% of ignorance which could be attributed to wiggling-related motives in the Self-Other-ignorance condition, underscoring the crucial importance of our within-subject approach.

As a further strength of our study, we linked decision and ignorance patterns to individual difference measures indicative of different dispositional motivations. Moderate (as opposed to higher or lower) prosociality predicted the behavioral pattern plausibly reflecting exploitation of moral wiggle room, corroborating Grossman and van der Weele’s^8^ findings that those individuals who are neither firmly prosocial nor selfish are most likely to engage in behavior that can be interpreted as wiggling. Prosociality as measured by SVO also negatively predicted ignorance in the Self-Other and No-Tradeoff contexts but not in the Other-Other context, suggesting that selfish orientations could drive ignorance in tradeoff situations involving personal stakes. The Other-Other context highlighted complexities in prosocial behavior. Sixteen percent of participants ignored exclusively in this context, and these individuals exhibited high dispositional prosociality. This unexpected finding suggests that competing prosocial considerations, such as fairness versus efficiency, may drive ignorance when both options are equally defensible from a prosocial point-of-view.

Despite the strengths of our within-subject approach, our study has limitations. First, selective attrition was observed, with dropouts exhibiting lower prosociality and conscientiousness. Second, time effects emerged, as participants were less likely to ignore in later waves. As conditions were counterbalanced across waves, these factors did not bias the primary findings. However, they may account for the lower overall ignorance (22.52%) and slightly elevated baseline prosociality (76.91%) observed, relative to Dana et al.^5^. Given that we replicated Dana et al.’s^5^ results in a pre-study using the same participant pool (see Supplemental Materials), these deviations likely reflect design-specific influences rather than sample composition. Consequently, while the absolute prevalence rates may not generalize, the observed behavioral patterns suggest that inattention and tradeoff aversion, alongside self-interest, play a role in willful ignorance.

Third, instead of directly measuring motivations, in our experimental approach we designed decision contexts such that they render specific kinds of motivation less plausible as drivers of ignorance. Thus, we inferred plausible motivations from patterns of behavior across contexts. Importantly, the decision contexts differed qualitatively: In the Self–Other context, participants chose between a selfish and a prosocial option; in the Other–Other context, they chose between an efficient and a fair option; and in the No-Tradeoff context, one option was always strictly dominant. These qualitative differences prevent a direct comparison of allocation decisions between decision contexts. Accordingly, we focused, on the one hand, on comparisons of ignorance choices, and, on the other hand, on how the option to ignore affected allocation decisions (i.e. within-person decision shifts). Still, we need to discuss whether each decision context might have introduced further context-specific motivations that could plausibly play into and account for ignorance decisions in the respective context. For example, we cannot rule out the possibility that in the Other-Other context, specifically, an individual’s preference for efficiency, could have motivated ignorance. A clear preference for efficiency would render the hidden information irrelevant, so that allocation choices should be unaffected by the option to remain ignorant. However, this is not what we observed: also in the Other-Other context, allocation choices were affected by the option to ignore. Yet, potentially pointing to another context-specific motivation, we observed that some participants chose to ignore only in the Other–Other context (and not in Self-Other or No-Tradeoff contexts). This group was characterized by higher dispositional prosociality compared to other groups, suggesting that ignorance might serve a specific purpose in this context—for example, delegating the choice between efficiency and fairness to chance, which can be seen as a specific type of tradeoff aversion. To the extent that such context-specific motivations affected ignorance choices, our quantifications of allocation and ignorance types can only be considered an approximation for the respective plausible relevance of wiggling-related motives, tradeoff aversion and inattention as motivations for willful ignorance. We believe that, despite this limitation, our within-subject approach provides insight into how multiple factors may plausibly contribute to ignorance in prosocial decision making, as operationalized by the classic hidden information treatment by Dana et al.^5^. For future research, we believe that investing into further pinpointing the motivations driving ignorance in different decision contexts will be highly fruitful.

Generally, while standard accounts assume that people should always seek to acquire more information^1^, especially when it is instrumental to their decision making^2^, it is important to note that ignorance cannot automatically be regarded as irrational or maladaptive. In fact, ignorance can serve as an adaptive strategy that helps individuals manage emotional overload, reduce cognitive load, and maintain strategic flexibility. Different forms of ignorance may fulfill different adaptive functions. For instance, wiggling-related ignorance can protect against punishment^30^, tradeoff aversion can facilitate emotion regulation^20,21^, and inattention may reflect the prioritization of other information, or the avoidance of misleading information^31^. Viewed through this lens, the motivations underlying ignorance should be context dependent and can be seen as reflecting rational, adaptive responses to specific circumstances.

Beyond willful ignorance in prosocial decision making, there are several other domains in which people deliberately avoid information (for reviews, see^2,32^). Although our study primarily addresses willful ignorance in prosocial contexts, our findings may also serve as a reminder that caution is warranted when interpreting ignorance behavior more generally as a means to a particular end. Willful ignorance is potentially driven by a complex combination of diverse motivations.

In conclusion, the present research advances our understanding of willful ignorance in prosocial decision making by examining behavioral patterns across diverse contexts and inferring the plausible underlying motivations that might drive willful ignorance. Across these contexts, behavioral patterns are consistent with the interpretation that ignorance does not stem only from wiggling-related selfishness but also from discomfort with tradeoffs and inattentiveness. Effective interventions could address these varied potential drivers, combining strategies to simplify information, reduce cognitive load, and enhance salience while fostering genuine concern for others’ welfare. By elucidating the interplay of self-interest, prosocial considerations, and contextual influences, this study contributes to the broader discourse on ethical decision making and prosocial behavior.

## Materials and methods

### Data availability and transparency statement

All data, materials, analysis scripts, and pre-registration of design, hypotheses, and statistical analyses for this study are available at osf.io/p3a2g.

Note that two experimental conditions are not reported in this paper, but we report all results in the Supplemental Materials. In the results, we partly report different analyses than the pre-registered ones for illustrative purposes. All analyses also hold when using the pre-registered models. For all pre-registered analyses including pre-registered exploratory analyses, see Supplemental Materials.

This study was approved by the Ethics Council of the Max Planck Society within the framework of the Generalized Approval of Experiments Following the Protocol that is Standard in Experimental Economics (approval no. 2018_3 / 2021_36). The corresponding IRB renewal confirmation was issued on October 10, 2022. All methods were carried out in accordance with relevant guidelines and regulations. For all employed measurement instruments, our use was fully within the scope permitted by the applicable copyright regulations.

In March 2022, we collected data from 878 participants via Amazon Mechanical Turk (53.48% identifying as female) over the course of four waves (starting sample wave 1: N = 1110, 20% attrition). Written informed consent was obtained from all subjects prior to their participation in the study. We excluded data from participants who failed the attention check (n = 3), the control questions (n = 4) in wave 1, and those who did not participate in the subsequent wave within 48 h (n = 225). Participants failing the control questions in wave 2 to 4 were excluded from making a decision in the respective wave, but were allowed to participate in the following waves.

### Design, procedure, and incentivization

In wave 1, all participants filled out a questionnaire with dispositional measures. For an overview of all dispositional measures, see Supplemental Materials. In a pre-study, we conceptually replicated the results of Dana et al.^5^ in an online setting using charities as recipients, confirming that the setup was suitable for our purposes. For a detailed description and results, see Supplemental Materials. In wave 1 to 4, participants faced eight different allocation decisions (over the course of 10 days). We counterbalanced the order of the allocation decisions. In the first wave, after completing the dispositional measures, participants made their decision in the No-Tradeoff baseline condition. In the following three waves, participants always made one Self-Other context decision and one Other-Other context decision. In the fourth wave, participants eventually made their decision in the No-Tradeoff ignorance condition. Participants earned an average of $6.98 and donated $4.75. See Supplemental Materials for further details on the methods, including the two conditions we did not include in this paper and for all analyses on carry-over effects.

## Supplementary Information

Below is the link to the electronic supplementary material.

Supplementary Material 1

## Data Availability

For all data, analysis scripts, pre-registration and materials see osf.io/p3a2g.
