# Supplementary material for: Evidence for multiple motivational accounts of willful ignorance in prosocial decision making beyond moral wiggling
Source: Sci Rep. 2026 Jul 3;16:20528. doi: 10.1038/s41598-026-59730-1 (PMC13332247; doi:10.1038/s41598-026-59730-1)
Supplement: Supplementary file 1 — Supplementary Material 1 [file 41598_2026_59730_MOESM1_ESM.docx]

**Supplemental Materials**

for the article

***Willful Ignorance in Prosocial Decision Making is Driven by Moral Wiggling, Tradeoff Aversion, and Inattention***

Fiona tho Pesch^1,2^*, Anna Baumert^2,3^ & Susann Fiedler^2,4^

**Affiliations:**

^1^ Department of Psychology, University of Cologne; Cologne, Germany

^2^ Max Planck Institute for Research on Collective Goods; Bonn, Germany

^3^ Department of Social and Human Sciences, University of Wuppertal; Wuppertal, Germany

^4^ Department of Strategy and Innovation, Institute for Cognition and Behavior, ​Vienna University of Economics and Business; Vienna, Austria

* corresponding author: [fiona.thopesch@uni-koeln.de](mailto:fiona.tho.pesch@uni-koeln.de)

**Supplementary Table S1**

*All dispositional measures administered in the study (wave 1)*

| **Measure** | **Citation** | **Scale** | **Number of items** | **Example item** | **Internal consistency (McDonald’s omega)** | **Note** |
| --- | --- | --- | --- | --- | --- | --- |
| **Wiggling-related** | | | | | | |
| Social Value Orientation | Murphy et al., 2011 | n.a. | 15 | n.a. |  |  |
| Brief HEXACO Inventory - Honesty-Humility | De Vries, 2013 | 1 (strongly disagree) to 5 (strongly agree) | 4 | “I would like to know how to make lots of money in a dishonest manner.” | .642 |  |
| Guilt And Shame Proneness scale | Cohen et al., 2011 | 1 (Very unlikely) to 7 (Very likely) | 8 | “You lie to people but they never find out about it. What is the likelihood that you would feel terrible about the lies you told?” | .853 | Guilt subscale only |
| Brief Fear of Negative Evaluation Scale | Leary, 1983 | 1 (Not at all characteristic of me) to 5 (Extremely characteristic of me) | 8 | “I am afraid that others will not approve of me.” | .957 | Original scale has 12 items, item selection based on face validity and correlations. |
| **Tradeoff aversion** | | | | | | |
| Need for Closure | Kruglanski et al., 1993 | 1 (completely disagree) to 5 (completely agree) | 6 | “When I am confronted with a problem, I’m dying to reach a solution very quickly. ” | .879 | Decisiveness facet only |
| Desirability of Control | Burger & Cooper, 1979 | 1 (Doesn't apply to me at all) to 7 (Always applies to me) | 5 | “I enjoy having control over my own destiny.” | .777 | Original scale has 20 items, item selection based on face value and factor loadings. |
| **Inattention** | | | | | | |
| Need to Evaluate Scale | Jarvis & Petty, 1996 | 1 (extremely uncharacteristic of me) to 5 (extremely characteristic of me) | 16 | “I form opinions about everything.” | .887 |  |
| Brief HEXACO Inventory - Conscientiousness | De Vries, 2013 | 1 (strongly disagree) to 5 (strongly agree) | 4 | “I work very precisely.” | .647 |  |

##

## Carry-over and time effects, missing values and selectivity in dropouts

As pre-registered, we checked for order effects in a total of 13 Chi^2^-tests (one per behavioral outcome). We found no significant effects between our counterbalanced groups, all *p*s > critical *p* (Bonferroni corrected), and thus used the whole dataset. We also checked for time effects in the Self-Other and the Other-Other context decisions, as we counterbalanced the order in which participants were exposed to these contexts over the four data collection waves. Indeed, we found that the participants were less likely to ignore in later waves compared to earlier waves, OR = 0.74, 95% CI [0.65, 0.84]. We did not find time effects in participants’ allocation decisions, OR = 1.05, 95% CI [0.98, 1.12].

We employed Little’s test for randomness in missing values (Little, 1988), and found no suspicious pattern, Chi^2^(10) = 7.501, *p* = .678. As noted above, we had a total dropout of about 20% from wave 1 to wave 4, meaning that 878 of the initial 1110 participants took part in all four data collection waves. We explored differences between participants who dropped out and participants who took part in all four waves. When comparing participants who took part in all four waves to participants who dropped out at some point, we found significant differences in all dispositional measures except for SVO, with dropout participants showing lower scores on Honesty-Humility, Guilt Proneness, Desirability of Control, and Conscientiousness, but higher scores in the Need to Evaluate, Need for Closure, and the Brief Fear of Negative Evaluation, all *p*s < . 024.

# Further results

## Dispositional measures

**Supplementary Table S2**

*Intercorrelation table of dispositional measures*

|  | Honesty- Humility | Guilt Prone | BFNE | Closure | Control | Evaluate | Conscientious |
| --- | --- | --- | --- | --- | --- | --- | --- |
| SVO | **.173** | **.189** | -.046 | **-.084** | **.082** | **-.085** | -.002 |
| Honesty- Humility |  | **.434** | **-.188** | **-.219** | **.325** | **-.127** | **.272** |
| Guilt Prone |  |  | -.014 | **-.076** | **.220** | .049 | **.244** |
| BFNE |  |  |  | **.418** | **-.490** | -.022 | **-.315** |
| Closure |  |  |  |  | **-.459** | **.092** | **-.245** |
| Control |  |  |  |  |  | **.176** | **.505** |
| Evaluate |  |  |  |  |  |  | **.156** |

*Note:* Significant correlations (*p* < .05) in bold.

Abbreviations: Guilt Prone = Guilt Proneness; BFNE = Brief Fear of Negative Evaluation; Closure = Need for Closure; Control = Desirability of Control; Evaluate = Need to Evaluate

**Supplementary Table S3**

*Logistic regressions with dispositional measures predicting ignorance decisions*

|  | Log reg 1: Self-Other-ignorance | Log reg 2: Other-Other-ignorance | Log reg 3: No-Tradeoff-ignorance |
| --- | --- | --- | --- |
|  | OR  (*SE*) | OR  (*SE*) | OR  (*SE*) |
| SVO | **0.779****  (0.065) | 0.860  (0.074) | **0.761***  (0.084) |
| Honesty-Humility | **0.760****  (0.072) | 0.835  (0.082) | **0.693****  (0.083) |
| BFNE | 0.866  (0.090) | **0.786***  (0.084) | **0.625****  (0.093) |
| Guilt Proneness | **0.840***  (0.075) | 1.039  (0.100) | **0.805***  (0.088) |
| Control | 0.905  (0.105) | **0.739****  (0.087) | 0.799  (0.121) |
| Closure | 0.927  (0.094) | 1.029  (0.106) | 1.045  (0.142) |
| Evaluate | 0.948  (0.086) | 1.018  (0.095) | 1.074  (0.132) |
| Conscientiousness | 1.071  (0.107) | 1.038  (0.106) | 1.143  (0.152) |
| Constante | **0.271****  (0.023) | 0.247  (0.022) | **0.128***  (0.016) |
| *N* | 864 | 864 | 734 |
| Pseudo R^2^ | .043 | .025 | .078 |

*Note.* Standard errors in parentheses. * *p* < .05, ** *p* < .01, ***, *p* < .001

Abbreviations: BFNE = Brief Fear of Negative Evaluation; Closure = Need for Closure; Control = Desirability of Control; Evaluate = Need to Evaluate

**Supplementary Table S4**

*Regressions with dispositional measures predicting selfish/efficient/antisocial decisions*

|  | Log reg 1a: Self-Other- baseline | Log reg 1b: Self-Other- ignorance | Log reg 2a: Other-Other- baseline | Log reg 2b: Other-Other- ignorance | Log reg 3a: No-Tradeoff- baseline | Log reg 3b: No-Tradeoff- ignorance |
| --- | --- | --- | --- | --- | --- | --- |
|  | OR (*SE*) | OR (*SE*) | OR (*SE*) | OR (*SE*) | OR (*SE*) | OR (*SE*) |
| SVO | **0.335*****  (0.032) | **0.422*****  (0.049) | **0.850***  (0.062) | 0.871  (0.087) | **0.645***  (0.128) | 0.881  (0.155) |
| Honesty- Humility | 0.867  (0.090) | **0.704****  (0.092) | 0.984  (0.084) | **0.797***  (0.092) | 0.806  (0.159) | 0.773  (0.143) |
| BFNE | 0.993  (0.111) | 0.828  (0.120) | 1.058  (0.092) | 0.826  (0.094) | 0.664  (0.172) | 0.622  (0.156) |
| Guilt Proneness | 0.829  (0.084) | **0.747***  (0.090) | 0.864  (0.070) | 1.060  (0.123) | 1.012  (0.207) | 0.776  (0.126) |
| Control | 0.896  (0.112) | 1.001  (0.152) | 1.010  (0.100) | 1.100  (0.142) | **0.537***  (0.144) | **0.462****  (0.115) |
| Closure | 0.871  (0.095) | 0.869  (0.113) | 0.860  (0.074) | 1.122  (0.132) | 0.975  (0.238) | 0.692  (0.170) |
| Evaluate | 0.940  (0.092) | 0.946  (0.116) | 0.980  (0.075) | 0.929  (0.101) | 1.500  (0.346) | 1.205  (0.245) |
| Conscientiousness | 1.193  (0.131) | 1.035  (0.132) | 0.931  (0.079) | 0.936  (0.105) | 0.911  (0.209) | 1.200  (0.254) |
| Constante | **0.215*****  (0.022) | **0.480*****  (0.055) | **0.528*****  (0.038) | 0.907  (0.086) | **0.023*****  (0.006) | **0.081*****  (0.017) |
| N | 873 | 448 | 873 | 456 | 866 | 393 |
| Pseudo R^2^ | .201 | .170 | .014 | .017 | .091 | .105 |

*Note.* Standard errors in parentheses. * *p* < .05, ** *p* < .01, ***, *p* < .001

### Ignorance types and dispositional measures

Investigating potential driving factors of the different patterns of ignorance we run ANOVAs testing for differences between the four different ignorance types in inter-individual difference scores. We excluded participants who did not fall into any of these four types. The analysis revealed differences in SVO scores, *F*(4, 675) = , *p* < .001 between the individual ignorance types. Specifically, participants who consistently *revealed* had a higher SVO score than *wiggling ignorers*, *t*(549) = 4.20, *p* < .001. *Only-OO ignorers* also shows higher SVO scores than *wiggling ignorers*, *t*(106) = -2.64, *p* = .010. Similarly, types differed in terms of their scores on Honesty-Humility, *F*(4, 675) = 5.48, *p* < .001. Again, consistently revealing participants had higher average scores than *wiggling ignorers*, *t*(549) = 2.01, *p* = .045, *tradeoff ignorers*, *t*(512) = 3.05, *p* = .002, and *consistent ignorers*, *t*(537) = 3.35, *p* < .001. *Only-OO ignorers* showed significantly higher Honesty-Humility scores than *tradeoff ignorers*, *t*(69) = -2.46, *p* = .016, as well as *consistent ignorers*, *t*(94) = -2.52, *p* = .013. These differences in dispositional prosociality is also mirrored in different behavior of the *Only-OO ignorers* in the Self-Other baseline condition: They are significantly more likely to choose the prosocial option compared to *wiggling ignorers*, *χ²*(1) = 5.63, *p* = .018, as well as compared to *tradeoff ignorers*, *χ²*(1) = 6.04, *p* = .014, but not *consistent ignorers*, *χ²*(1) = 1.57, *p* = .210.

We furthermore saw differences in Guilt Proneness between the types, *F*(4, 675) = 4.50, *p* < .001. Notably, participants who consistently revealed showed higher Guilt Proneness scores than *wiggling ignorers*, *t*(549) = 3.62, *p* < .001, and *consistent ignorers*, t(537) = 2.48, p = .014. *Only-OO ignorers* had higher scores than *wiggling ignorers*, *t*(106) = -2.84, *p* = .005, and *consistent ignorers*, *t*(94) = -2.14, *p* = .035. There were no differences between types in the Brief Fear of Negative Evaluation, Desirability of Control, Need for Closure, Need to Evaluate or Conscientiousness, all *p*s > .05.

### Allocation types and dispositional measures

Using an ANOVA, there was a significant difference between the three groups of *consistent prosocials*, *consistent selfish* and *moral wigglers* in terms of their SVO scores, *F*(2, 429) = 71.05, *p* < .001. Participants classified as *moral wigglers* showed lower SVO scores compared to the consistent prosocials, *t*(345) = 4.04, *p* < .001, but higher SVO score than the consistently selfish participants, *t*(159) = -5.81, *p* < .001.^^[[1]](#footnote-1)^^ The same pattern showed for Guilt Proneness, all *p*s < .001. We also saw differences between these groups with regards to Honesty-Humility: Consistent prosocials showed higher scores than moral wigglers, *t*(345) = 4.89, *p* < .001, but there was no significant difference between consistent selfish types and moral wigglers, *t*(159) = 1.20, *p* = .231. We found no significant differences between these groups in the Brief Fear of Negative Evaluation scale, Desirability of Control, Need for Closure, Need to Evaluate, or Conscientiousness, all *p*s > .44.

## Post-decision questionnaire

In the post-decision questionnaire, participants perceived the decision in the Other-Other-baseline as compared to the Self-Other-baseline to be more bothersome, *t*(870) = -5.25, *p* < .001, more troublesome, *t*(868) = -6.82, *p* < .001, and they felt more conflicted, *t*(869) = -6.61, *p* < .001, and guilty, *t*(869) = -4.76, *p* < .001. Participants reported to be more interested in the decision in the Self-Other-baseline compared to the Other-Other-baseline, *t*(872) = 2.56, *p* = .011, but not more motivated to make the decision, *t*(872) = 0.22, *p* = .826. Participants in the No-Tradeoff-baseline reported lower values on all these questions except for the question about how motivated they were to make the decision compared to the Self-Other-baseline, all *p*s < .003. These ratings in the baselines were not related to ignorance decisions in the respective ignorance conditions later on, all *p*s > .05.

# Pre-study

The pre-study’s main aim was to replicate the effect of Dana et al.’s (2007) hidden information treatment on prosociality within an online context using charities as recipients. Furthermore, we wanted to identify two charities that would be similar to one another in terms of popularity.

## Methods

We recruited 100 mTurk participants through CloudResearch. Two participants failed the comprehension questions in the beginning of the experiment and were screened out. After passing two sets of comprehension questions, we asked participants to make the incentivized allocation decision, distributing money between themselves and the charity “Direct Relief” (see Figure S1). We randomly assigned participants either to a baseline condition, in which all payoffs were visible, or to an ignorance condition. In the baseline condition, participants faced the decision of receiving a bonus payment of $0.60 while donating $0.10 (option A), or receiving only $0.50 while donating also $0.50 (option B). In the ignorance condition, the payoffs to the charity were hidden behind question marks. Participants could reveal the payoffs for the charity by clicking a “Reveal”-button. Participants then either faced aligned payoffs, meaning that option A is better for both the participant ($0.60) and the charity ($0.50); or unaligned payoffs, meaning that option A is better for the participant ($0.60), but worse for the charity ($0.10). After participants made their decision, we asked them a set of post-decision questions about their emotional state. Lastly, we asked participants to rate five different charities on four items in terms of their popularity.

**Supplementary Figure S1**

*Screenshot of the ignorance condition*


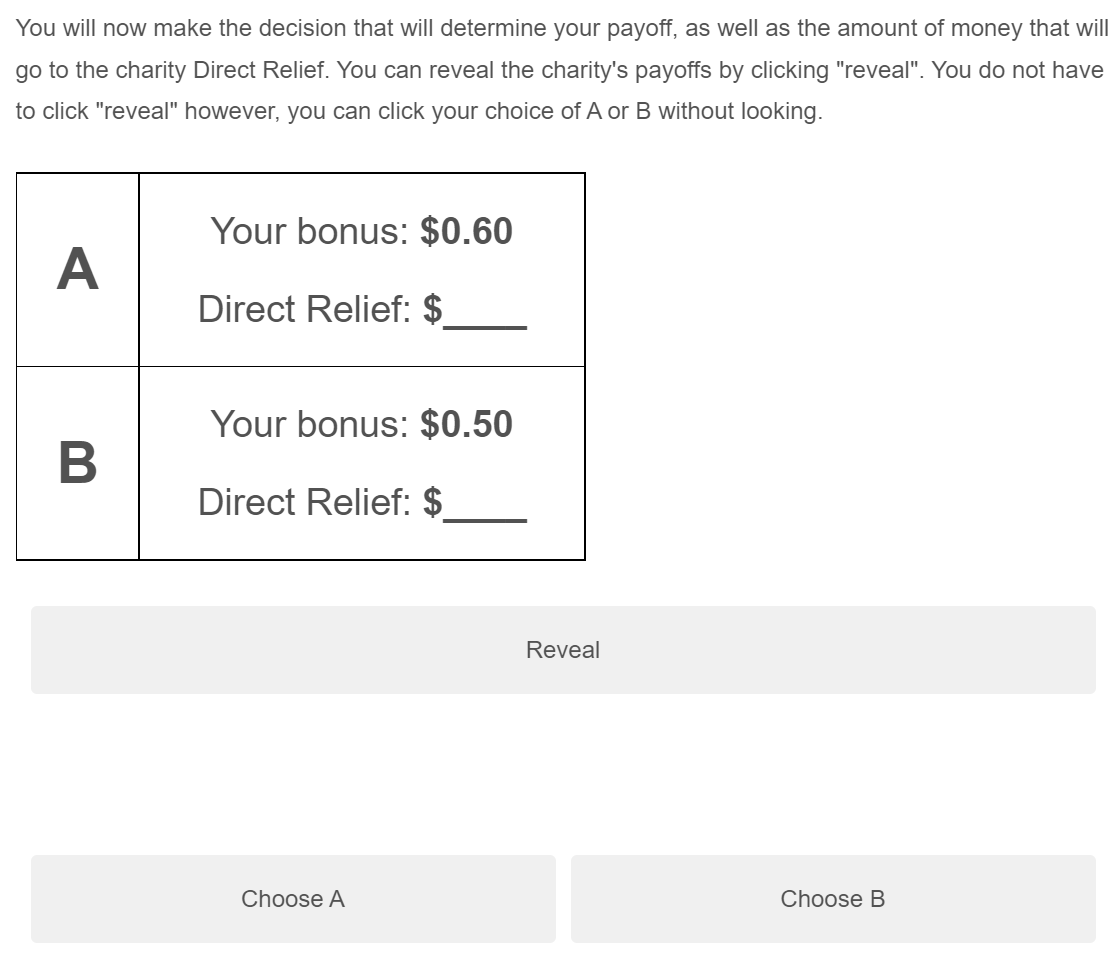


## Results

Out data reveal a significant main effect of our manipulation on prosocial behavior: participants in the baseline condition are more likely to choose the prosocial option (69.4%) compared to participants in the ignorance condition (34.4%), Chi^2^(1) = 8.363, *p* = .004, Cramer’s V = -.351. On average, 56.5% of participants ignore the information on the consequences of their behavior if they can.

We did not find any significant differences between our experimental conditions in the post-decision questionnaire: Participants in the ignorance condition experienced the same amount of decision conflict, *t*(96) = -0.351, *p* = .726, and reported similar degrees of state guilt, *t*(96) = -0.200, *p* = .905, than participants in the baseline, and we did not observe any differences in terms of people’s self-image, *t*(96) = 0.029, *p* = .977.

**Supplementary Table S5**

*Summary of the behavioral results of our study*

| Condition | Share of decisions for option B | Share of ignorance decisions |
| --- | --- | --- |
| Baseline (n = 36) | 69.4% | - |
| Ignorance unaligned (n = 32) | 34.4% | 50.0% |
| Ignorance aligned (n = 30) | 13.3% | 63.3% |

### Charity evaluations

The two charities with the highest reported likelihood to donate to are the “American Red Cross” (*M* = 3.55, *SD* = 1.13) and “Feeding America” (*M* = 3.60, *SD* = 1.07). As the ratings for these two charities also had considerably high correlations on all four items (correlations between .39 and .61), we decided to use them as charities in our Other-Other contexts.

## Discussion

We successfully replicated the findings of Dana et al. (2007)’s hidden information treatment. Prosocial behavior dropped from around 70% in the baseline treatment to about 35% in the ignorance condition with unaligned payoffs. To compare: in the original study, there were 74% of participants who chose the prosocial option in the baseline condition, and 37% in the ignorance condition with unaligned payoffs. In our study, only 43.5% of participants chose to reveal the true payoffs. In the original study, this proportion was 56% of participants.

# Extended methods

## Design and procedure

In a within-subject design with four data collection waves spread over ten days, participants faced eight different allocation decisions in three Self-Other conditions (including the prosocial ignorance condition, see below), three Other-Other conditions (including the fair ignorance condition, see below), and two No-Tradeoff conditions. Five of these contexts also involved the decision of whether or not to ignore. We counterbalanced the order in which participants faced the eight different allocation decisions among three counterbalancing groups (see Table S2). In the first wave, all participants answered to a battery of dispositional measures, and then made their decision in the baseline condition of the No-Tradeoff context. In the following three waves, participants always made one of the three Self-Other context decisions before being exposed to an Other-Other context. In the fourth wave, participants eventually made their decision in the ignorance condition of the No-Tradeoff context. After each baseline condition, participants answered a short post-decision questionnaire about how they felt when making the decision.

Participants were informed that the first survey was part of a series of four surveys that would be sent over the next ten days. On the first page of the survey, we asked participants only to take the survey if they were willing to participate in the following three surveys. The surveys were sent out at three-day intervals, and participants had 48 hours to complete the survey after receiving it. To minimize attrition we offered a $3 bonus payment for participants who completed all four surveys. Participants were informed that the first survey would take approximately 20 minutes to complete, while the other three surveys would take about 6 minutes each.

### Incentivation

Participants earned an average of $6.98 and donated $4.75 on average throughout the four waves of data collection. In the first wave, participants earned a flat fee of $0.70, and received additional bonus payment according to one randomly drawn incentivized SVO decision. In wave two to three, participants received a flat fee of $0.10, and a decision-contingent bonus payment of between $0.50 and $0.60. In all waves, donations were for the charities Feeding America, The American Red Cross, and Direct Relief.

**Supplementary Table S6**

Counterbalanced order of eight decisions context for the three counterbalancing groups

|  | 1. Group  (Self-Other-baseline) | 2. Group  (selfish ignorance) | 3. Group  (prosocial ignorance) |
| --- | --- | --- | --- |
| Wave 1 | Dispositions  No-Tradeoff-baseline | Dispositions  No-Tradeoff-baseline | Dispositions  No-Tradeoff-baseline |
| Wave 2 | Self-Other-baseline  Other-Other-fair ignorance | Self-Other-selfish ignorance  Other-Other-baseline | Self-Other-prosocial ignorance  Other-Other-efficient ignorance |
| Wave 3 | Self-Other-selfish ignorance  Other-Other-efficient ignorance | Self-Other-prosocial ignorance  Other-Other-fair ignorance | Self-Other-baseline  Other-Other-baseline |
| Wave 4 | Self-Other-prosocial ignorance  Other-Other-baseline  No-Tradeoff-ignorance | Self-Other-baseline  Other-Other-efficient ignorance  No-Tradeoff-ignorance | Self-Other-selfish ignorance  Other-Other-fair ignorance  No-Tradeoff-ignorance |

## Material

### Decision contexts

Participants faced eight different allocation decisions in three different decision contexts: (1) The Self-Other contexts, (2) the Other-Other contexts, and (3) No-Tradeoff contexts. In all decision contexts, participants made a binary allocation decision between two options, A and B.

#### Self-Other context

In the Self-Other context, participants chose how to distribute money between themselves and the charity “Direct Relief”. In the Self-Other-*baseline condition*, all payoffs were visible when choosing between a selfish option (A) and a prosocial option (B). Option A would result in a payoff of $0.60 for the recipient, and a donation of $0.10. Option B would mean $0.50 for both the participant and the charity. In the *selfish ignorance condition* (which corresponds to the Self-Other-ignorance condition in our paper), participants faced the same options, but the payoffs of the charity were initially hidden. Participants had the option of either choosing option A or B without knowing the donation attached to these options, or revealing the payoffs of the charity before the decision. Participants were informed that it would be randomly decided whether participants would face unaligned payoffs (i.e., the payoff structure of the Self-Other-baseline condition), or aligned payoffs (i.e., a payoff structure in which the payoffs for the charity would be flipped). In the latter case, option A would be better for both the participant and the charity. In the *prosocial ignorance condition*, option A was the prosocial option, while option B was the selfish option. This time, the payoffs of the participants were hidden. Again, participants could decide for either option without knowing the payoffs to themselves or reveal the payoffs. Participants were told they would be randomly assigned to either aligned or unaligned payoffs.

**Supplementary Figure S2**

*Schematic representation of the decision context in the Self-Other-baseline, the selfish ignorance condition (which corresponds to the Self-Other-ignorance condition in our paper), and the prosocial ignorance condition*

*
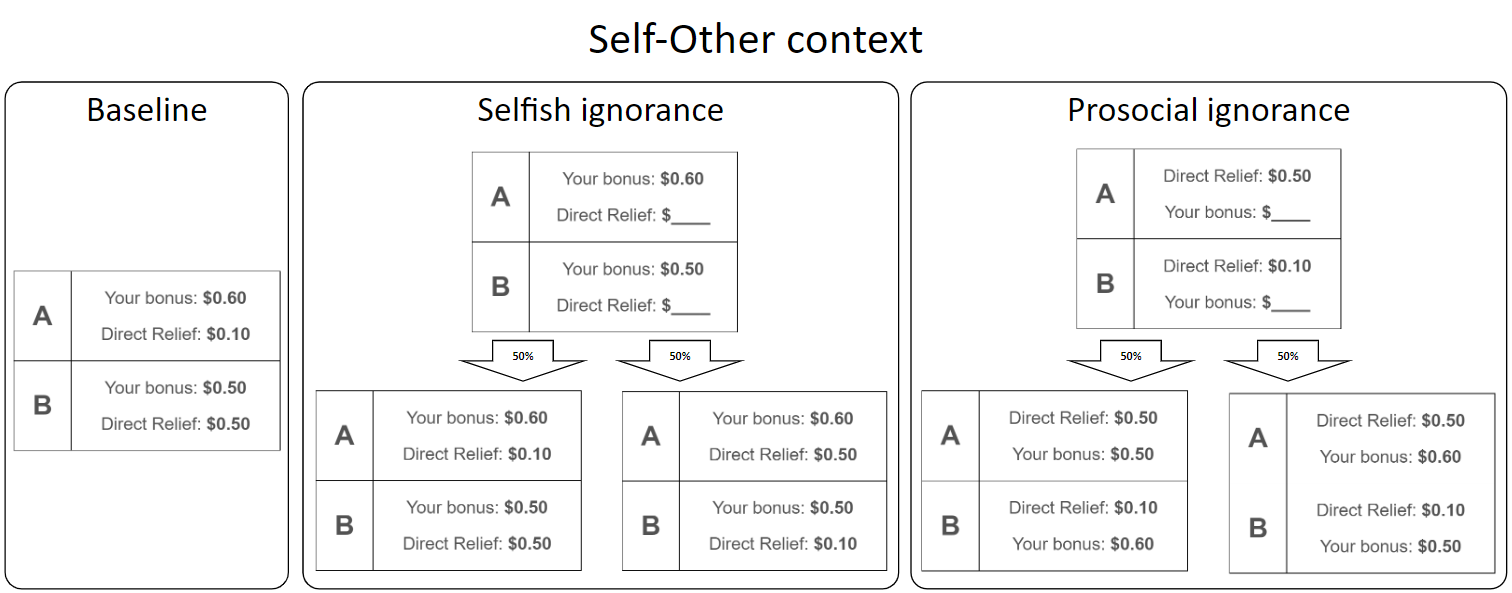
*

#### Other-Other context

In the Other-Other context, participants distributed money between the two charities “American Red Cross'' and “Feeding America”, facing a tradeoff between a fair and an efficient option. We counterbalanced between subjects which charity would be associated with option A or B. In the Other-Other-*baseline condition*, participants could choose between an efficient option (A) and a fair option (B). Option A would result in a donation of $0.60 to charity 1, and a donation of $0.10 to charity 2. Option B would mean $0.30 for each charity. In the *efficient ignorance condition* (which corresponds to the Other-Other-ignorance condition in our paper), payoffs of the charity with the lower payoffs were hidden. Similar to the Self-Other context, the payoffs of both charities were aligned in half of the cases and unaligned in the other half. In the *fair ignorance condition*, payoffs of the charity with the higher payoffs were hidden. In this condition, option A was the fair option, while option B was the efficient option. In both the efficient and the fair ignorance condition, participants could decide for either option without knowing the full payoff distribution, or reveal the payoffs by clicking a “reveal” button.

**Supplementary Figure S3**

*Schematic representation of the decision context in the Other-Other-baseline, the efficient ignorance condition (which corresponds to the Self-Other-ignorance condition in our paper), and the fair ignorance condition.*

*
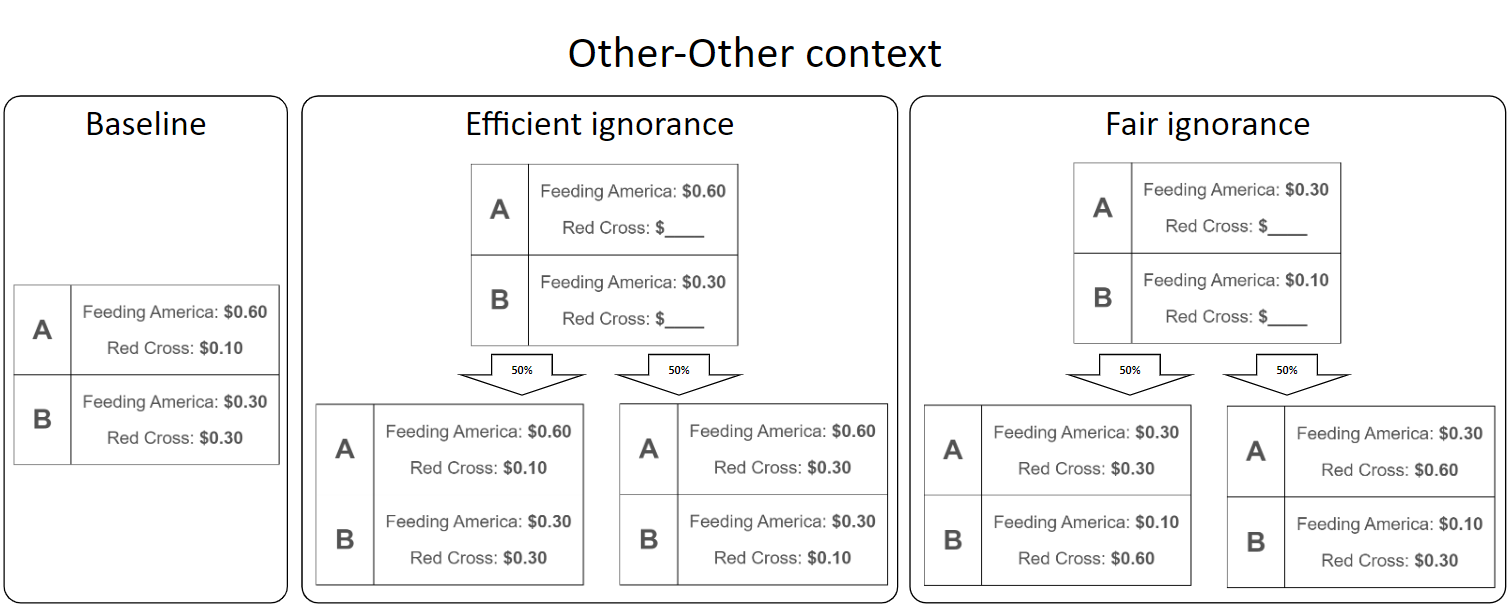
*

#### No-Tradeoff context

In the No-Tradeoff context, participants again distributed money between the two charities, “American Red Cross'' and “Feeding America”, counterbalancing between subjects which charity would be associated with option A or B. In the No-Tradeoff-*baseline condition*, participants could choose between an antisocial option (A) and a fair and efficient option (B). Option A would result in a donation of $0.50 to charity 1, and a donation of $0.10 to charity 2. Option B would mean $0.50 for each charity. In the *No-Tradeoff-ignorance condition*, payoffs to one charity were hidden, so that participants did not know which option was the antisocial option. Again, participants could inform themselves about the full payoff structure by clicking a “reveal” button.

**Supplementary Figure S4**

*Schematic representation of the decision context in the No-Tradeoff-baseline and the No-Tradeoff-ignorance condition*


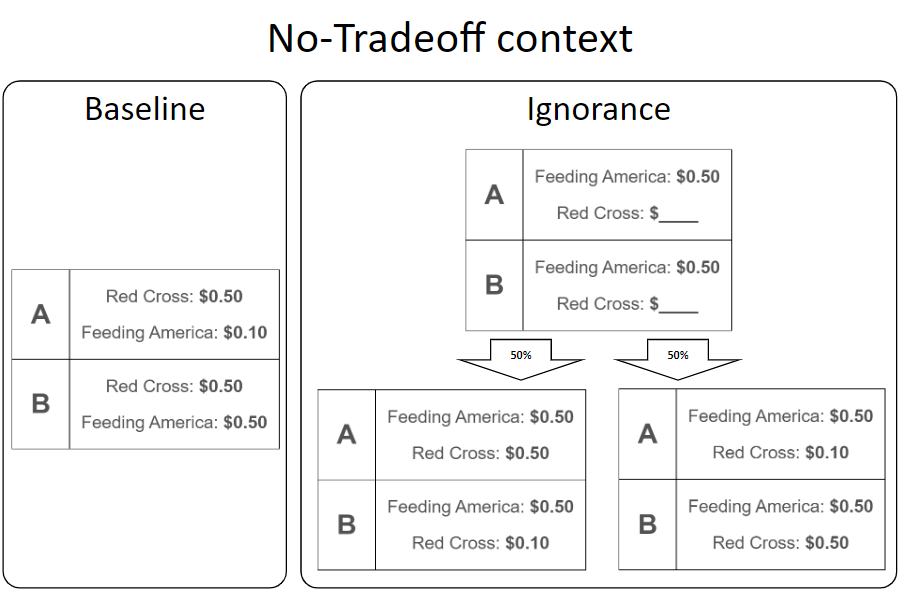


### Rationale for excluding prosocial and fair ignorance from our main analysis

For the eight conditions participants made their decisions in, we only include six in the main manuscript. We decided to exclude the Self-Other prosocial ignorance condition, as well as the Other-Other fair ignorance condition for different reasons.

The prosocial ignorance condition has already been employed in prior research (Kandul & Ritov, 2017; Moradi, 2018). This research already indicated that people also ignore within this context, however, it does not change behavior. We set out to replicate this finding using a larger sample. As this condition does not fit into our main story of investigating potential alternative mechanisms behind the observed behavior in the classic hidden information treatment of Dana et al. (2007), we did not include it in the main manuscript. For all results on the prosocial ignorance condition, see section “Prosocial ignorance” below.

With regard to the fair ignorance condition, we originally planned to collapse the Other-Other fair and the Other-Other efficient ignorance condition into one Other-Other-ignorance condition (see pre-registration). Unfortunately, we could not do so, because option A represents the efficient option in the Other-Other-baseline, but the fair option in the fair ignorance condition. Though we re-coded behavior from the fair ignorance condition in a way that option A represents the efficient option, collapsing behavior from the fair and Other-Other-ignorance condition would mean equating fair decisions in the fair ignorance condition with efficient decisions in the efficient ignorance condition. As the efficient ignorance condition is coded in the same direction as the Other-Other-baseline (option A = efficient option), and thus is equivalent to the selfish (option A = selfish option) and the No-Tradeoff-ignorance conditions (option A = antisocial option), we used the efficient ignorance condition for our main analyses. In that way, the fair ignorance condition was comparable to the prosocial ignorance condition, in which decisions were also coded in the opposite direction of the respective baseline. For all results on the fair ignorance condition, see section “Fair ignorance” below.

### Typologies

Utilizing the within-subject manipulation of our study design, we can identify exactly who ignores in which context, and how this affects their allocation decisions. We came up with two typologies. The first typology identified who engaged in moral wiggling (i.e., allocation types): We categorized participants into moral wigglers, consistent prosocials and consistent selfish types by comparing their allocation decisionsin the Self-Other-baseline with their allocation decisionsin the selfish ignorance conditions. The second typology determines different ignorance types, categorizing participants into consistent revealers, *wiggling ignorers*, *tradeoff ignorers* and *inattentives*. Consistent revealers did not engage in ignorance in any of the three ignorance conditions. *Wiggling ignorers* are participants who ignored exclusively in the selfish ignorance condition. *Tradeoff ignorers* ignored both in the selfish and the efficient ignorance condition, while *inattentives* ignored in all three ignorance conditions. Finally, we can combine both typologies to see which type of ignorers engaged in ignorance within the selfish ignorance condition, as well as which type of ignorers were most likely to engage in moral wiggling as defined above.

### Dispositional measures

In wave 1, all participants filled out a questionnaire with eight dispositional measures. We measured SVO using the slider measure (Murphy et al., 2011), which involves a series of 15 scenarios, in which participants have to make dictator game decisions, distributing money between themselves and another participant. We furthermore used 8 items from the Brief Fear of Negative Evaluation Scale (Leary, 1983), which were selected based on intercorrelations and face validity. We used the decisiveness facet (6 items) of the Need for Closure scale (Kruglanski et al., 1993) as our measure for dispositional tradeoff aversion. To gauge people’s dispositional tendency to be inattentive, we used the 16-item Need to Evaluate Scale (Jarvis & Petty, 1996). For an overview over all dispositional measures, see Table S3.

#### Rationales for dispositional measures

We further complemented our exploration of the motivational mechanisms of (patterns of) behavior, by linking differences in behavioral patterns across conditions with measures of dispositional characteristics. Specifically, we identified dispositional concepts that capture individual differences in those motivations, and how to measure them.

With regards to the motivation of *wiggling*, people’s dispositional tendencies to behave prosocially or selfishly are connected to willful ignorance. It has been proposed that it is people with moderate prosocial tendencies who exploit moral wiggle room, meaning that they are not pronounced prosocials, but also not extremely selfish (Grossman & van der Weele, 2017). As people who ignore are not all wiggling, but potentially also consistent selfish participants who are not interested in the hidden information, we would assume that the more dispositionally selfish a person, the more likely they are to ignore. For wiggling behavior itself, we would expect people with a moderate prosocial disposition to engage in moral wiggling. Social Value Orientation (SVO), which is a concept measuring stable preferences for joint outcomes and cooperation (prosocial values) as compared to a pro-self orientation (P. Van Lange, 1999), has been shown to reliably predict prosocial behavior (Balliet et al., 2009; Murphy et al., 2011; Smith, 2012; Van Lange et al., 2007; see (Thielmann et al., 2020 for a review).

The motivation of *tradeoff aversion* describes a reluctance to make tradeoff decisions. People differ in how much they value making their own decisions (Beattie et al., 1994). These interindividual differences can be captured by the concept of Need for Cognitive Closure (Webster & Kruglanski, 1994). People higher (vs. lower) in Need for Closure tend to take decisions quickly (if necessary) and avoid them (if possible; Kruglanski, 1989; Kruglanski & Webster, 1996). A study relying on vignettes suggests that people who are high in Need for Closure are also more likely to be decision averse (Otto et al., 2016).

The motivational factor of *inattention* captures the extent to which people pay attention to a situation. It can be conceptually linked to the Need to Evaluate (Jarvis & Petty, 1996). The concept captures a personality trait that reflects a person’s proclivity to create and hold attitudes (Bizer et al., 2004). People high in Need to Evaluate are especially likely to form attitudes towards all sorts of objects. If willful ignorance is partly driven by any form of inattention, people who have a stronger attitude towards a topic should be less likely to ignore information due to inattention, as they should be more engaged in the decision.

Furthermore, we explored additional dispositional measures related to the three motivations above. The factor of wiggling can also be conceptually linked to image concerns, or how much people care about what others think of them. Image concerns have long been thought to drive wiggling (Adena & Huck, 2020; Andreoni & Bernheim, 2009; Grossman, 2015; Grossman & van der Weele, 2017). Image concerns can be operationalized by the Brief Fear of Negative Evaluation Scale (Reichenberger et al., 2015). The scale captures a person's tolerance for the possibility of being judged negatively by others. Furthermore, there are alternative ways of measuring selfishness. Instead of using SVO as a measure for selfishness, we could also utilize the factor Honesty-Humility of the HEXACO model (Ashton & Lee, 2007), or the factor Guilt Proneness of the Guilt and Shame Proneness scale (Cohen et al., 2011) to capture people’s propensity to engage in prosocial behaviors (see Thielmann et al., 2020 for a review). Another way to capture tradeoff aversion is the Desirability of Control scale (Burger & Cooper, 1979), while inattention could also be captured by the factor Conscientiousness of the HEXACO model (Ashton & Lee, 2007). We thus explored the relationship of our behavioral outcomes with these dispositional measures to be able to identify which concept is best at capturing the underlying motives of behavior.

# Prosocial ignorance

The *prosocial ignorance condition* was part of the Self-Other context. Allocation decisions in this condition were compared to decisions in the Self-Other-baseline condition in order to test whether prosocial decisions increased when ignorance was possible. In the prosocial ignorance condition, option A was the prosocial option, while option B was the selfish option. While in the selfish ignorance condition payoffs to the charity were hidden, this time the payoffs of the participants themselves were hidden. Again, participants could decide for either option without knowing the payoffs to themselves or reveal the payoffs. Participants were told they would be randomly assigned to either aligned or unaligned payoffs.

## Results

In order to see in which way the option to ignore potentially can also increase prosociality, we investigated decision behavior of participants in the prosocial ignorance condition.

Running a repeated measurement logistic regression with ignorance condition (prosocial vs. selfish) predicting ignorance decisions, there was no significant difference in the proportion of people who ignored in the prosocial (22.57%) and the selfish (22.52%) ignorance condition, OR = 1.00, 95% CI [0.78, 1.29].

Comparing allocation decisions between prosocial ignorance condition and Self-Other-baseline, we ran a repeated measurement logistic regression with condition (Self-Other-baseline vs. prosocial ignorance) predicting allocation decision. Participants were not more likely to select the prosocial option in the prosocial ignorance condition (79.12%) compared to the Self-Other-baseline condition (76.91%), OR = 0.97 95% CI [0.86, 1.10].

When investigating ignorance types in the Self-Other context, the majority of participants consistently revealed the information in both the selfish and the prosocial ignorance condition (63.50%). While 13.97% of all participants ignored only in the selfish ignorance condition, 14.08% ignored only in the prosocial ignorance condition; 8.45% ignored in both the selfish and the prosocial ignorance condition.

Looking at how decision behavior differs between the Self-Other-baseline and the prosocial ignorance condition showed that 72.41% of all participants consistently chose the prosocial option, while 14.35% consistently chose the selfish option. 6.62% of participants chose the selfish option in the Self-Other-baseline, and the prosocial option in the prosocial ignorance condition (i.e., *prosocial wigglers*), while 6.62% of participants showed the reverse pattern.

Of those participants who ignored the prosocial ignorance condition, the majority (74.07%) were consistently prosocials, and only a small minority (7.41%) chose the selfish option in the Self-Other-baseline, but the prosocial option in the prosocial ignorance condition. The remaining 18.52% chose the option with the lower payoff under ignorance, which was not compatible with any of our hypothesized motivations.

Using logistic regressions with all dispositional measures predicting prosocial ignorance, only the Brief Fear of Negative Evaluation scale predicted ignorance decisions.

# Fair ignorance

The fair ignorance condition is based on the Other-Other-baseline condition, in which participants could choose between an efficient option (A) and a fair option (B). Option A would result in a donation of $0.60 to charity 1, and a donation of $0.10 to charity 2. Option B would mean $0.30 for each charity. In the *fair ignorance condition*, payoffs of the charity with the higher payoffs were hidden. In this condition, option A was the fair option, while option B was the efficient option. This meant that the fair option was the one with the higher payoff under ignorance. Participants could decide for either option without knowing the full payoff distribution, or reveal the payoffs by clicking a “reveal” button.

## Results

First, we investigated the effect of having the option to ignore on allocation decisions. Our results showed that participants were not significantly more likely to select the fair option in the fair ignorance condition (69.59%), compared to the Other-Other-baseline (65.26%), OR = 0.95, 95% CI [0.89, 1.01]. When looking at how many people ignored, we saw that ignorance levels did not significantly differ between the efficient (20.44%) and fair ignorance condition (18.31%), OR = 0.79, 95% CI [0.57, 1.09]. In terms of dispositional measures, fair ignorance was predicted by Honesty-Humility, as well as the Brief Fear of Negative Evaluation scale.

We can furthermore investigate different ignorance types. In the Other-Other context, the majority of participants consistently revealed the information in both conditions (72.22%). While 9.50% of all participants exclusively ignored in the Other-Other-ignorance condition, 7.62% only ignored in the fair ignorance condition. The remaining 10.67% ignored in both the efficient and the fair ignorance condition.

To sum up, though we observed similar levels of ignorance in the fair as compared to the Other-Other-ignorance condition, we did not observe a significant change in allocation decision. As such, similar to the prosocial ignorance condition, ignorance did not impact subsequent behavior, as people who ignore in the fair ignorance condition chose the fair option in both the Other-Other-baseline and the fair ignorance condition.

# Pre-registered results

In the following, we report analyses as pre-registered (<https://osf.io/p3a2g/?view_only=531aa932f3d94fe49bbc6447fba3cde1>). Unfortunately, we cannot collapse data from the two Other-Other contexts, as the baseline for these two conditions have to be reversed-coded, because there are more participants preferring the efficient option in the Other-Other-baseline. Thus, we only include the Other-Other-ignorance condition in the analyses on decision behavior reported here, but results also hold when including the fair ignorance condition instead.

We first pre-registered to investigate potential carry-over or order effects by looking at whether the condition has a significant effect on behavior for each of the behavioral responses individually. As this results in 13 different analyses (8 allocation decisions, 5 ignorance decisions), we adjusted our p-level accordingly using the Bonferroni correction method (critical p-value: 0.05/13=0.003846). None of the analyses passed the critical p-value, thus we include data from all four waves of data collection in our analyses.

**H1: Participants facing unaligned payoffs in the ignorance condition (compared to baseline condition) are more likely to choose the option with the higher payoff under ignorance (or in the No-Tradeoff condition the option with the lower efficiency).**

Our data supports this hypothesis, OR = 2.043, z = 8.36, *p* < .001: While participants choose the option with the higher payoff under ignorance in 20.413% in the baseline conditions, this share increases to 32.077% in the ignorance conditions.

**H2: There is an interaction of the factors ignorance and decision context on choice behavior: The impact of the factor ignorance on behavior decreases from selfish, to the Other-Other, to the No-Tradeoff condition.**

We find a significant interaction effect between the efficient and the No-Tradeoff-ignorance conditions, OR = 1.982, z = 2.23, p = .026. There is no interaction effect between the selfish and the Other-Other-ignorance condition, OR = 1.117, z = .55, p = .583.

**H3a: Participants will be more likely to ignore in the selfish, compared to the Other-Other-ignorance condition.**

Our data does not support this hypothesis, OR = 1.276, z = 1.48, p = .138. Participants in the Self-Other-ignorance condition are similarly likely to ignore (22.517%) compared to participants in the Other-Other-ignorance conditions (20.439%).

**H3b: Participants will be more likely to ignore in the Other-Other-, compared to the No-Tradeoff-ignorance condition.**

We find that participants are significantly more likely to ignore in the Other-Other- compared (20.439%) to the No-Tradeoff-ignorance condition (13.451%), OR = 0.360, z = -5.17, p < .001.

**H4a: SVO predicts ignorance in the Self-Other-ignorance condition better than in the other ignorance conditions.**

We find that SVO is not a better predictor for ignorance in the selfish, as compared to the Other-Other-ignorance conditions, OR = 1.021, z = 1.70, p = .090, or the No-Tradeoff-ignorance condition, OR = 0.994, z = -.41, p = .683.

**H4b: Need for Closure predicts ignorance in the Self-Other-ignorance and Other-Other-ignorance conditions better than in the No-Tradeoff-ignorance condition.**

Need for Closure does not predict ignorance better the the selfish, OR = 0.935, z = -.34, p = .737, or in the Other-Other-ignorance conditions, OR = 1.195, z = .89, p = .373, compared to the No-Tradeoff-ignorance condition.

**H4c: Need to Evaluate predicts ignorance across all ignorance conditions.**

Need to Evaluate does not predict ignorance across all ignorance conditions, OR = 1.179, z = .61, p = .544.

**Hp1: Participants facing unaligned payoffs are more likely to choose the prosocial option in the prosocial ignorance condition compared to the Self-Other-baseline condition.**

Participants facing unaligned payoffs in the prosocial ignorance condition are not more likely to choose the prosocial option compared to participants in the Self-Other-baseline condition, OR = 0.972, z = -.45, p = .655.

**Hp2: Participants are more likely to ignore in the prosocial compared to the Other-Other-ignorance condition.**

Participants are more likely to ignore in the prosocial (22.569%) compared to the Other-Other-ignorance conditions (19.375%), OR = 1.417, z = 2.62, p = .009.

**Hp3: SVO predicts ignorance in the prosocial ignorance condition better than in the Other-Other-ignorance and the No-Tradeoff-ignorance condition.**

We find the hypothesized interaction effect of SVO and context in predicting ignorance for the prosocial and Other-Other context, OR = 0.961, z = -3.81, p < .001, as well as for the prosocial and the No-Tradeoff context, OR = 0.936, z = -4.74, p < .001. However, this is due to the fact that in the prosocial ignorance condition, SVO is (insignificantly) positively related to ignorance, OR = 1.010, z = 1.60, p = .109, while SVO is negatively related to ignorance in all other conditions.

**He1: Dispositional guilt predicts prosocial decisions in the Self-Other-baseline, but not in the Self-Other-ignorance condition when payoffs are unaligned.**

There is no interaction effect of dispositional guilt and condition in predicting prosocial decisions, OR = 1.426, z = 1.67, p = .096.

**He2: SVO-conflict predicts people's propensity to switch within the Self-Other conditions.**

SVO-conflict does not predict people’s propensity to switch within the Self-Other conditions, OR = 0.995, z = -.27, p = .789.

**He3: Efficiency-fairness-conflict predicts people's propensity to switch within the Other-Other-conditions.**

Efficiency-fairness-conflict does not predict people's propensity to switch within the Other-Other-condition, OR = 1.056, z = 1.42, p = .156.

**He4: Anticipated image damage and anticipated guilt in the Self-Other-baseline condition predict ignorance in the Self-Other-ignorance condition.**

Anticipated guilt in the Self-Other-baseline condition predicts ignorance in the Self-Other-ignorance condition, OR = 1.285, z = 2.67, p = .008.

**He5: Experienced conflict and experienced difficulty in the respective baseline conditions predict ignorance in the Self-Other and Other-Other conditions respectively.**

Within the Self-Other condition, there is no relationship between ignorance and experienced conflict, OR = 1.024, z = .23, p = .815, or experienced difficulty, OR = 1.315, z = 1.83, p = .067, respectively.

Within the Other-Other-condition, there is a relationship between ignorance and experienced conflict, beta = -.030, t = -2.24, p = .025, and experienced difficulty, beta = .044, t = 2.39, p = .017, respectively.

**He6: Participants scoring high on laziness in the baseline conditions are more likely to ignore in the respective ignorance conditions.**

Laziness-scores of the baseline predicted ignorance in the Self-Other context, OR = 0.847, z = -2.04, p = .041, but not in the Other-Other context, beta = -.005, t = -.45, p = .653, or the No-Tradeoff context, OR = 0.873, z = -1.40, p = .163.

1. Note that these analyses resemble the results of Grossman and van der Weele (2017) on the relationship between SVO and willful ignorance. However, they used engaging in ignorance as a proxy for exploiting moral wiggle room, as they did not employ a within-subject design. We can also replicate their results directly, showing that participants who ignored in the selfish ignorance condition had higher SVO scores than participants who revealed, but behaved selfishly, *t*(167) = 3.02, *p* = .003, but a lower SVO-score compared to participants who revealed and chose the prosocial option, *t*(365) = -6.17, *p* < .001. [↑](#footnote-ref-1)
